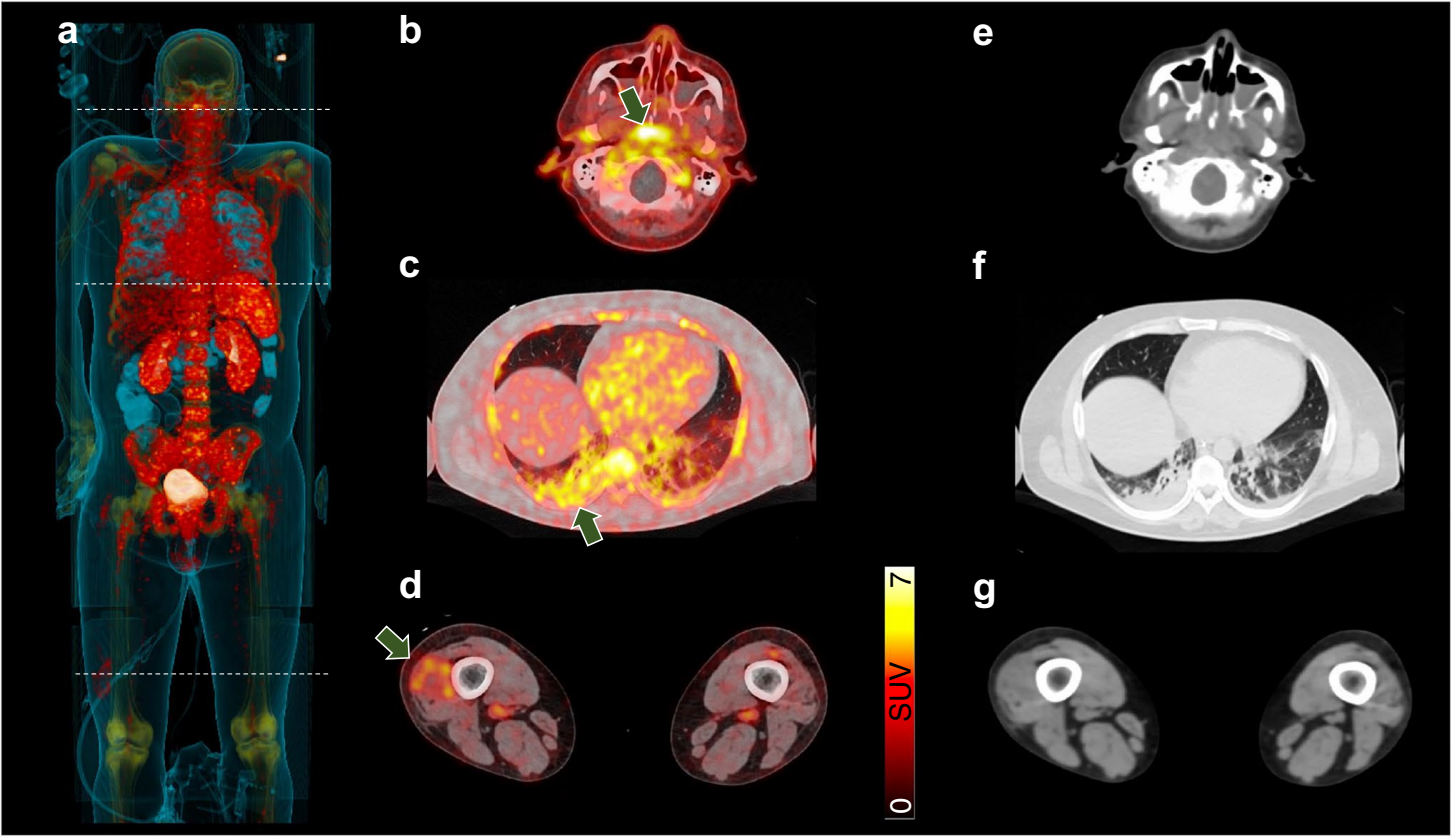# CXCR4-targeted molecular imaging after severe SARS-Cov-2 infection

**DOI:** 10.1007/s00259-022-05932-4

**Published:** 2022-08-12

**Authors:** Alessandro Lambertini, Philipp E. Hartrampf, Takahiro Higuchi, Sebastian E. Serfling, Patrick Meybohm, Andreas Schirbel, Andreas K. Buck, Rudolf A. Werner

**Affiliations:** 1 Department of Nuclear Medicine, University Hospital Würzburg, Oberdürrbacher Str. 6, 97080 Würzburg, Germany; 2 Faculty of Medicine, Dentistry and Pharmaceutical Sciences, Okayama University, Okayama, Japan; 3 Department of Anaesthesiology, Intensive Care, Emergency and Pain Medicine, University Hospital Wuerzburg, Würzburg, Germany; 4 Division of Nuclear Medicine and Molecular Imaging, The Russell H Morgan Department of Radiology and Radiological Sciences, Johns Hopkins School of Medicine, Baltimore, MD USA

We herein report on a 37-year-old male which suffered from severe pneumonia requiring mechanical ventilation due to acute infection with severe acute respiratory syndrome coronavirus type 2 (SARS-CoV-2; COVID-19). Despite optimal anti-inflammatory treatment, the patient’s condition further deteriorated, leading to elevated inflammatory blood-based biomarkers, septic shock, and persistent temperature above 40 °C. Inflammatory-directed, whole-body positron emission tomography/computed tomography (PET/CT) targeting C-X-C motif chemokine receptor 4 (CXCR4) was scheduled to identify sites of inflammation, including the lung and distant sites of disease. In addition, previous studies investigating neutrophils also rendered CXCR4 as a potential target in severe cases [1]. After injection of CXCR4-targeting [^68^Ga]Ga-PentixaFor, increased radiotracer accumulation was noted in the bone marrow and spleen on maximum intensity projection (a), indicating hematopoietic activation. Additional CXCR4-expressing, inflammatory foci were identified on transaxial PET/CT in the pharyngeal/palatine tonsils (b), along with right-dominant bilateral pneumonia (c), and in the distal right thigh (d, green arrows), which may be partially explained by intramuscular inflammation. On transaxial CT (e–g), only pulmonary involvement was recorded.

There is increasing evidence on a multisystem inflammatory component in the context of SARS-CoV-2 [2] and thus, a PET-based whole-body read-out may provide relevant information on the current status quo. In this regard, [^18^F]FDG PET has already been applied in an acute setting after SARS-CoV-2 infection, but its challenging image protocol including prolonged fasting may hamper its more widespread adoption for emergency cases [3], in particular in patients that require glucose containing medication on intensive care units. The CXCR4-targeting radiotracer [^68^Ga]Ga-PentixaFor, however, has already been used in various infectious and inflammatory scenarios [4–6], as it allows to visualize CXCR4 expression on infiltrating leukocytes [7]. Relative to [^18^F]FDG, no further patient preparation is needed, thereby providing fast implementation in clinical routine [8]. Of note, chemokines are crucially involved in the inflammatory immune response after respiratory SARS-CoV-2 infection, in particular in severe cases. In those high-risk individuals, CD10^Low^CD101^−^ CXCR4^+^ neutrophils have been identified, with a significant accumulation in blood and lungs [1], in a manner similar to the herein presented case (a, c). In addition, [^68^Ga]Ga-PentixaFor also revealed distant inflammatory foci in the thigh (d) and thus, CXCR4-targeted PET may also allow to provide a read-out of chemokine-induced hyperinflammation even in distant sites of disease. Differential diagnosis of this uptake, however, may also include accidental injury caused by daily patient care. Further studies to determine the intensity of radiotracer uptake relative to severity of the disease, the impact on patient management, the predictive potential of the PET signal for adverse outcome, or PET-based assessment of organ crosstalk (e.g., between the spleen and foci) are warranted. Such investigations, however, should not be limited to [^68^Ga]Ga-PentixaFor, but also include other inflammatory-targeted radiotracers, which also does not require challenging patient preparation [9].